# Factors governing the performance of Auxiliary Nurse Midwives in India: A study in Pune district

**DOI:** 10.1371/journal.pone.0226831

**Published:** 2019-12-27

**Authors:** Thidar Pyone, Shilpa Karvande, Somasundari Gopalakrishnan, Vidula Purohit, Sarah Nelson, Subha Sri Balakrishnan, Nerges Mistry, Matthews Mathai

**Affiliations:** 1 Centre for Maternal and Newborn Health, Liverpool School of Tropical Medicine, Liverpool, United Kingdom; 2 The Foundation for Research in Community Health, Aundh, Pune, Maharashtra, India; ESIC Medical College & PGIMSR, INDIA

## Abstract

**Background:**

The Auxiliary nurse midwife (ANM) cadre was created to focus on maternal and child health. ANMs are respected members of their communities and established providers of maternal and child health care within the community and at the facility level. Over time, additional roles and responsibilities have been added. Despite the importance of ANMs in the primary healthcare system in India, studies that consider factors governing the performance of ANMs in their workplaces are limited. We aimed to study factors governing performance of ANMs in Pune district, India.

**Methods:**

Semi-structured interviews were conducted with 13 purposely selected key informants at facility, district, state, and national levels. Focus group discussions were conducted with 41 ANMs and 25 members of the community. Non-participatory observations with eight ANMs provided information to expand on and scrutinise findings that emerged from the other lines of inquiry. A realist lens was applied to identify ANMs’ performance as a result of “mechanisms” (training, supervision, accountability mechanisms) within the given “context” (regulatory system, infrastructure and resources, ANMs’ expanded scope of work, gender roles and norms).

**Results:**

Weak enforcement of regulatory system led to poor standardisation of training quality among training institutions. Challenges in internal accountability mechanisms governing ANMs within the health system hierarchy made it difficult to ensure individual accountability. Training and supervision received were inadequate to address current responsibilities. The supervisory approach focused on comparing information in periodic reports against expected outputs. Clinical support in workplaces was insufficient, with very little problem identification and solving.

**Conclusion:**

Focusing on the tasks of ANMs with technical inputs alone is insufficient to achieve the full potential of ANMs in a changing context. Systematic efforts tackling factors governing ANMs in their workplaces can produce a useful cadre, that can play an important role in achieving universal health coverage in India.

## Background

The Auxiliary Nurse Midwife (ANM) cadre in India was created in the 1950s to focus on basic maternal health including midwifery and child health within the first two years of life [1]. In 1966, the Mukherjee Committee recommended inclusion of family planning under the Maternal and Child Health (MCH) programme [2]. Subsequently, the Government of India added additional functions and integrated these with MCH [2]. In 1975, ANMs became designated as multipurpose workers (MPW) that were required to provide child health services and primary curative care to the communities [1]. However male MPWs who mainly focussed on water and sanitation and some vertical programmes were difficult to recruit. Hence, implementing national health programmes gradually became the main responsibilities of ANMs [3]. Thus the scope of work of ANMs broadened to cover family planning, immunisation, infectious disease prevention and care, in addition to care during pregnancy and childbirth. Additionally, ANMs were responsible for supporting other activities such as mobilising eligible couples for sterilisation, accompanying mobilised couples to sterilisation camps and attending these camps [3,4].

**Fig 1:**
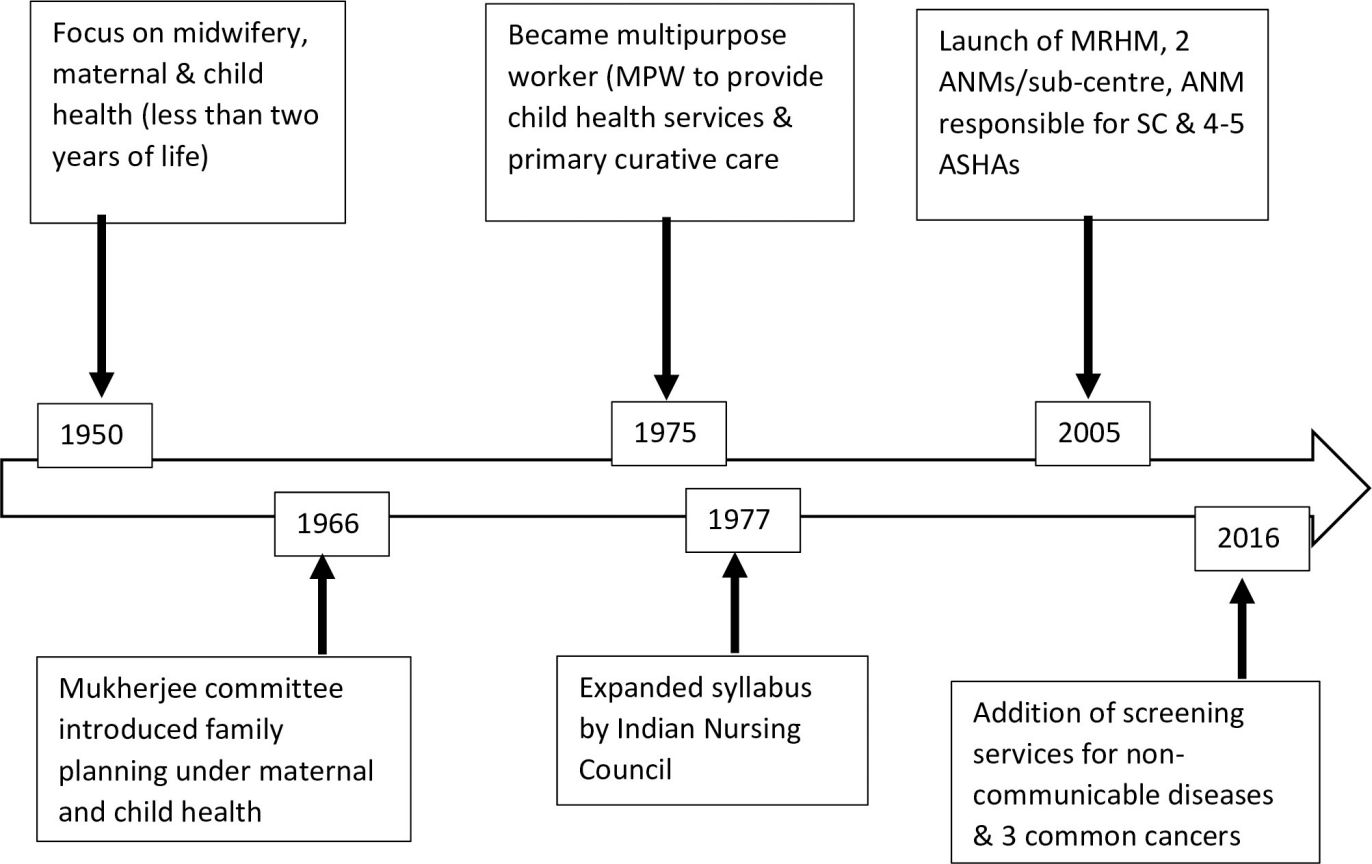
The historical context.

In 2005, with the introduction of the National Rural Health Mission (NRHM), ANMs were recognised as essential frontline health workers [2]. The NRHM introduced the posting of two ANMs at sub-centres to carry out midwifery related responsibilities which had been neglected [2]. ANMs then became responsible for managing sub-centre strengthening funds and for the supervision of female community health workers called Accredited Social Health Activists (ASHA). Changes in roles and responsibilities of ANMs (Fig 1) were further compounded by changes in the duration of ANM training from two years to 18 months with a reduction in the midwifery component since 1977 [1,3,4]. The duration of ANM training is currently 24 months inclusive of six months practical training.

Under the current National Health Mission (NHM), ANMs are placed at sub-centres (SC) catering to a population of 5,000 and at Primary Health Centres (PHCs) catering to a population of 30,000. Generally, ANMs are required to live at the sub-centres so that they can be on call for community members requiring their services. The provision of support and supportive supervision is irregular and many ANMs work in isolation [5,6].

**Fig 2:**
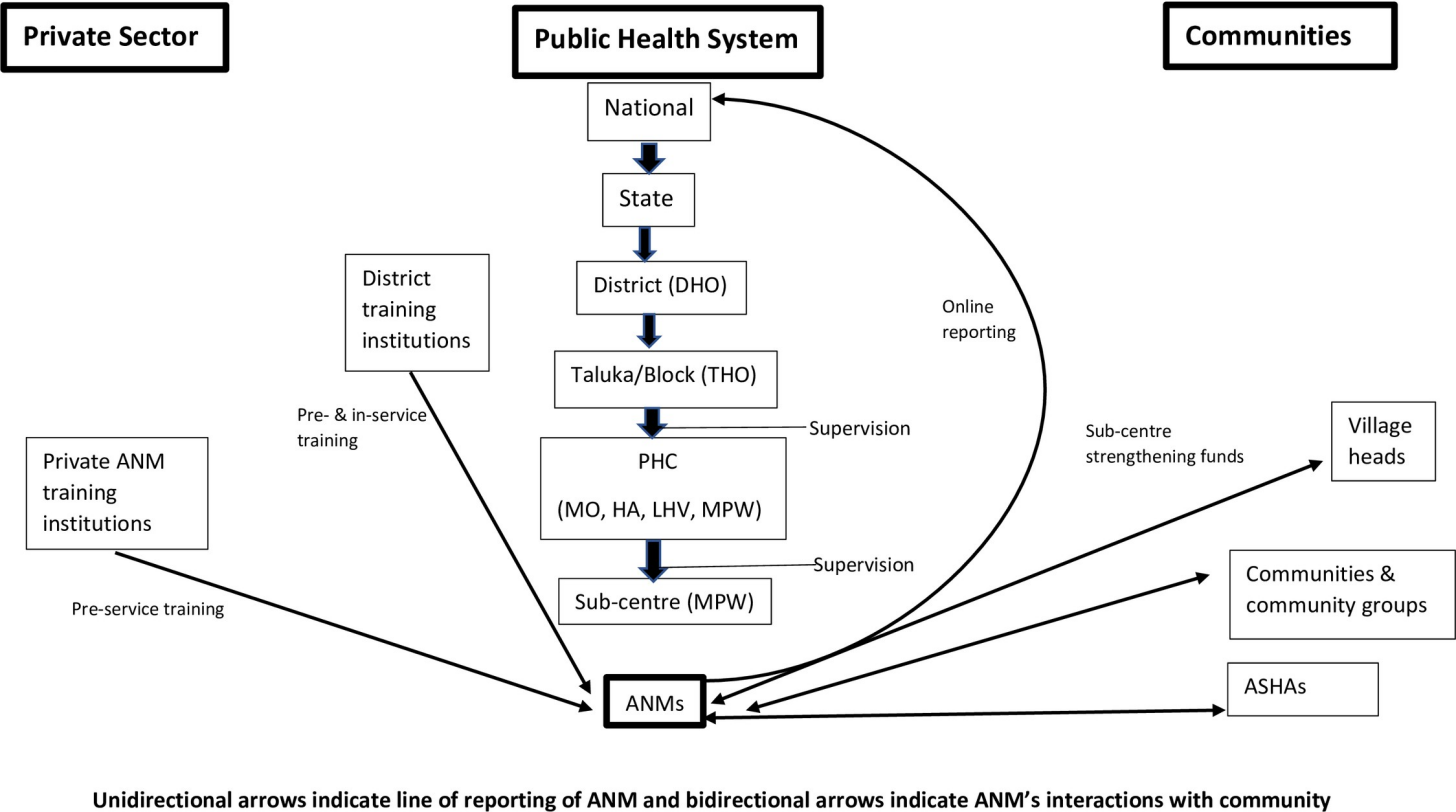
Summary map illustrating how an ANM is situated within the health system of India.

In the new era of Sustainable Development Goals, ANMs are an essential asset to the Indian health system in efforts to achieve universal access to sexual and reproductive healthcare services [7]. The ANM is well placed, as she is a respected member of her community and an established provider of maternal and child health care within the community and at the facility level. Fig 2 provides an overview of the current role of the ANM within India’s health system and highlights the different interfaces at the different levels and their key functions.

Recent economic developments in India have influenced the socio-demographic, epidemiological and health situation in the country. For instance, life expectancy has increased by 8.6 years for men and 10.6 years for women since the 1990s [8]. All states in India have had epidemiological transitions from communicable, maternal and neonatal disorders (CMND) to non-communicable disorders (NCDs) between 1990 and 2016 [8]. However, the existing pre-service syllabus issued by the Indian Nursing Council for ANMs includes most relevant clinical/technical areas except NCDs [9,10]. Thus it would appear that the training of ANMs may be inadequate to satisfactorily address the extended scope of work resulting from changes in the country’s epidemiological profile.

Given the importance of ANMs for the Indian health system and their ever-evolving role especially in the upcoming campaign of Ayushman Bharat in India [11], the rarity of empirical studies on ANMs from the wider health system perspective is surprising [12]. Particularly, studies that investigate factors governing the performance of ANMs at their workplaces are limited [13]. Factors influencing ANMs in their practices, scope of work and the mechanisms that influence ANMs’ performances remain understudied.

To address this gap, we aimed to explore factors governing ANMs’ performance that are crucial for the success of the cadre to support the achievement of universal health coverage in India. This study adds to the current knowledge on ANMs by applying a realist lens proposed by Pawson & Tilley and understanding ANMs’ performance as a result of “mechanisms” (training, supervision, accountability mechanisms) within a certain “context” (regulatory system, ANMs’ expanded scope of work, infrastructure and resources) [14]. We present our findings from a study in the Pune district of Maharashtra state, India between March and May 2017.

## Methodology

### Study context

Pune district is the fourth most populous district in India with a total population of 9,426,959 in 2011 [15]. It comprises two municipal corporations and 13 rural blocks (administrative units also known as talukas). Data for this study were collected in five of 13 rural blocks of Pune district.

### Study design and methods

This was a cross-sectional study using qualitative research methods. Semi-structured interviews with 13 purposely selected key informants (KIIs) (S1 Appendix) were conducted at their places of work in English by TP (female, MD, PhD, public health researcher based in Liverpool) at state and national levels and in Marathi (local language) by SK (female, PhD, public health researcher based in Pune) and VP (female, MSc, public health researcher based in Pune) at facility and district levels using semi-structured topic guides (S1 Guide). Each interview took 45 min on average.

FGDs of 60–90 min duration were conducted in Marathi by SK and VP with ANMs and community groups (S1 Appendix), using topic guides to discuss key areas of the ANMs programme (S2 Guide). Maximum variation sampling [16] was used to recruit ANMs from the selected administrative blocks with the aim of identifying central themes that emerged across the variety of cases. These administrative blocks were purposively selected for their differences in socio-economic and health accessibility characteristics. Sampling ensured that overall the FGDs included participants from: (i) the health facility furthest from the administrative centre; (ii) health facilities in tribal dominant area (relatively poor access); and (iii) easily accessible health facilities.

One FGD per ANM group was conducted at a health facility in each of the five blocks (Purandar, Ambegaon, Daund, Maval and Haveli). These five FGDs included 41 ANMs. A sixth FGD was conducted with ANMs who were attending skills training in the Government Skills Lab in Pune.

At the community level, two FGDs were conducted in community halls with a total of 25 women from Purandar and Maval block communities. Purposive sampling was used to select married community women aged between 20 and 30 years who were either pregnant or had at least one child below five years of age.

All participants provided written informed consent for FGDs and KIIs and for audio recording: there were no refusals. At the end of each KII and FGD, the research team (all external to the context) summarised key points for the participants. All FGDs were conducted in an environment where participants could express themselves comfortably. FGDs and all the KIIs in Pune were conducted in Marathi; whereas, the KIIs at the state and national levels were conducted in English. Interviews and FGDs conducted in Marathi were transcribed in Marathi and translated into English.

Non-participant observation of eight ANMs at their workplaces (conducted by TP) focused on three areas: (i) clinical skills; (ii) management and organisational skills; and (iii) record keeping and reporting skills (see Supporting Information). Only activities of ANMs that did not involve intimate procedures were observed. While observing ANMs’ activities in the field, the research team assumed “non-participant observer” roles, meaning that the team did not participate in the activities and discussions of ANMs, as the sole purpose was to observe ANMs at their work places. We also sought consent from the community participants who received services from the ANMs observed. Observations took 45–60 min and the observation data were recorded through note-taking (S3 Guide). In total, eight ANMs were observed at their workplaces. Data from the observations were used to triangulate/ corroborate and visualise the situations and challenge findings that emerged from KIIs and FGDs.

All transcribed data were anonymised and labelled according to the characteristics of participants. The transcribed FGDs and KIIs material were analysed thematically by the research team. Two researchers (TP and SN) first coded the data independently and compared and collated the codes systematically, using a realist lens. SK and VP then checked the initial coding frameworks produced by TP and SN. Transcripts and field notes were imported into MS Word and MS Excel for analysis. The data were coded initially using open codes which were then compared to the theoretical framework. After construction of a preliminary coding scheme, each code was examined in detail and further refined into categories.

A framework (composed of context, mechanisms and outcomes) was developed in which categories/sub-themes were allocated a column and used to code data. Each participant was allocated a row in the matrix. Thematic visual maps were generated using Mind Genius^®^ Business Version 6 to display key patterns and findings, by each of the main thematic areas (S2 Appendix). During this stage, the validity of the themes and whether they accurately represented the data were ensured among four researchers (TP, SN, SK, VP). Notes were taken during non-participant observation. They were further corroborated with findings from the FGDs and KIIs. We used qualitative content analysis [17]. A realist lens of Pawson and Tilley (context, mechanisms and outcomes) was applied for presenting results.

The study was approved by the ethics review boards of the Foundation for Research in Community Health (FRCH) (IREC-2017/22/02) and the Liverpool School of Tropical Medicine (LSTM) (17–012).

## Findings

By applying a realist lens, the study was able to unpack different factors that governed the performance of ANMs in their workplaces. The study identified “context” (regulatory system, ANM’s expanded scope of work, infrastructure and resources) and “mechanisms” (training, supervision, accountability mechanisms) that influenced the work of ANMs.

### Context

#### Regulatory system for ANM training institutions

**Lack of enforcement of regulations** compromised the quality of the ANMs’ pre-service training as some of the private institutions did not appear to be scrutinised by the existing regulatory system. This led to a **lack of standardisation of training quality and content among training institutions** (both public and private) that affected the quality of ANMs trained. In the words of a district-level ANM trainer:

“*We are given the syllabus in English*, *but they have not told us what to teach*. *What to teach in Anatomy*, *is not written anywhere*. *Suppose it is about the nervous system*, *from where should one take it*, *and if there are 10 hours for Anatomy*, *what important things I should teach them during those 10 hours*? *Our teachers refer Ross and Wilson or some other book and study it*. *They will prepare their own notes*.” (KI-6)

The national government was aware of the situation and finding ways to tackle the situation as commented by one national level participant:

*"They are not following all the protocols*, *or they don't know whether to give this drug or that drug and all those things*. *So*, *there are issues on that*, *the reason being that there is no standardisation of these ANM schools*. *You have some in the government*, *some in private and there is no standardisation*, *but we are working on that*.*"* (KI-11)

#### Infrastructure and resources

Another contextual factor that influenced ANMs was infrastructure and resources. ANMs faced operational challenges due to **lack of regular public transport**, **mobile network coverage and reliable supply of electricity, water and sanitation facilities** in the health facility. This finding was reported among ANMs from tribal areas where the health facilities (particularly sub-centres) were quite isolated with limited mobile network coverage. ANMs posted in remote villages faced challenges in conducting home visits as they were dependent on irregular and infrequent public transport. The following conversations of ANMs participating in an FGD illustrate this observation:

*“Respondent-2*: *this is a hilly area*, *madam*. *When I came first time to this PHC*, *(while climbing up) my leg got fractured and 2 to 2 ½ months I had a plaster*.

*Respondent-1*: *This place does not have transport facility*. *One vehicle comes in the morning and another comes at afternoon*. *At our sub-centre*, *bus reaches at 1 o’clock and it starts from here at 11 o'clock*. *I don’t get a bus to come back from there so when I go to home visit*, *I have to stay in some village*. *Here we do not have transportation facilities*.

*Respondent-1*: *Village is at on top of the hill*. *So*, *there is no transportation*. *Once we go there to visit then we have to come back in the evening*.".

Additionally, the lack of basic requirements such as water, electricity and toilets in the health facilities caused frustrations for ANMs and affected their motivation to work. This was significant for ANMs who were required to live at the sub-centres. The following quote from an ANM who participated in the FGDs illustrates this observation:

"*There is no water facility*. *At maximum places water is not available for consecutive eight days*, *a person needs to go for urination or latrine*, *then there is a problem*, *where they will go*, *no bathing facility*. *Why should only ANMs face all the problems*?"

#### Gender roles and norms

ANMs and some facility participants highlighted the challenges which ANMs faced because of gender roles and norms within their society. Some ANMs shared their concerns and how they did not feel physically or mentally safe at their workplaces as some of them were harassed by communities at their workplaces. Some facility participants also highlighted the lack of protection measures for ANMs at their workplaces. Most incidents were due to lack of security at their workplaces during the night or lack of protection measures within the system. In most cases, ANMs were harassed by some community people who were drunk or politically influential. Hence, ANMs noted that local leaders could be supportive by providing them secure workplaces within their communities when ANMs faced threats in their workplaces from community members. There were instances reported where ANMs were scolded for no reason but “due to political pressure in the presence of influential people”. The following quotes of facility participants exemplify this observation.

*“…there is a question of security*. *Many times*, *in PHC*, *people come drunk and at night we have a lot of problems*. *There is nobody like a security guard in the PHC*. *We have the ‘Mama’ [orderly]*. *But he is alone*. *There may be a patient for dressing*, *accident patient*, *our PHC is on the main road so once in 1–2 days such incidence happens*.” (KI-2)

"*Many times*, *it has happened here*. *I come here immediately since I stay close by at 1 km*. *Sister phones me and I reach here in half a second*. *We ‘handle it’ ourselves but there should be some ‘support’ that is what we feel*. *There is harassment from drunkards in the society… Other communities have these drunkards who would come at night and threaten you*, *“I am going to call such and such person* …*such and such political leader”*. *Our government is also such that*, *they will listen to them and in front of them they will scold us*, *this should not be done*. *They should first verify who is at fault*.” (KI-3)

Lady Health Visitors (LHVs) who supervise ANMs noted that support from politicians and local leaders were crucial to ensuring ANMs’ safety at their workplaces. While some ANMs received support from their communities, other ANMs had challenges in dealing with communities and those depended upon the interest of individual village leaders and the types of communities.

#### ANM’s expanded scope of work

Participants across different levels of the health system highlighted that the workloads of ANMs have increased significantly due to increase in the populations covered—the *“current ANM posts are based on the 2001 census*.” (KI-1). As per the national standard, one ANM in the non-tribal area is expected to cover a population of approximately 5,000 while those in the tribal areas cover populations of 3,000. The ANMs participating in the FGDs served populations of between 7,000 and 36,000. In addition, ANMs were responsible for entering beneficiaries’ information online which *“displaced caregiving so the time they should spend on looking at pregnant women went into looking at figures”* (KI-9).

The increased workload due to reporting was also confirmed during the research team’s observations, although it was based on one observation at an ANM’s workplace. The observation noted that the ANM retrospectively entered individual patient data from the preceding nine months. During the observation, the ANM confirmed that it was particularly important to avoid errors when entering the social security card number, mobile phone number and bank account number. Hence, the ANM had those numbers in her notebook. However, patients’ medical information and related data entered in the system, such as blood pressure, duration of pregnancy, haemoglobin level, etc were not based on available, verifiable records.

Some ANMs received support for data entry from their colleagues in the health facilities or district offices while others did not. Most of the time, family members of ANMs (husband or children) helped them with online data entry. Most ANMs struggled to accomplish those reporting tasks as they did not receive proper training on online reporting. In addition, ANMs struggled to familiarise themselves with the software that had evolved over time. One ANM noted that *“This software kept on changing*. *As soon as we learnt how to use it and started our data entry*, *it got changed*. *This is the 3rd software we are dealing with now*. *And the current RCH is really very complicated*.*”* (Observation ANM-1).

ANMs based at sub-centres or in PHCs with no internet access faced more challenges than those with access to the online reporting facilities (computer, electricity, and internet) as they had to travel solely for reporting purpose. In addition, there had been some occasions when ANMs were asked to provide urgently information online, leaving aside their health service delivery. One key informant from a health facility noted: *“There is an ongoing clinic and she [the ANM] receives a phone call from a higher level*, *“Give this report immediately”*, *she will pause her clinic work and complete this work [reporting]*. *So*, *there should be a separate data operator*, *only then ANM’s [technical] skill will be developed*.*"*(KI-3). Because of the increased workload, home visits have reduced significantly and these were covered mostly by ASHAs.

Participants across all levels of the health system acknowledged the increased workload of ANMs due to the increased population coverage, number of services and the online reporting. A national level participant suggested avoiding short-term solutions such as *"gap arrangements or ad hoc measures because that will actually create a serious problem later”* (KI-11). There have been efforts to reduce the reporting responsibilities of ANMs as stated by one of the district level participants:

"*A state-level study group is established to review and revise record keeping responsibilities of the ANMs*. *They are focusing on minimising the record keeping responsibilities of ANMs*. *In coming days*, *there will be some solution for it*.*"* (KI-1)

Nevertheless, according to national and district level participants, the Government of India has already planned to distribute tablet computers to ANMs for data entry. While this move would be expected to reduce the ANM’s workload by replacing manual data recording, ANMs will need additional training on online data entry.

Despite the burden of data collection on the ANMs’ workload, the extent to which reported data were analysed at higher levels and findings feedback to ANMs was not clear. None of the participants could clarify how the data had been used. One national level participant echoed this view as he felt it was an unnecessary collection of “*individual patient information*” which wasted resources at different levels of the health system. The same participant also noted that this was not the first study to highlight the fact that this “*individual or area-based reporting*” was reported to be not useful. He also highlighted that it was not clear how these data had been used as he had not observed any improvement in efficiency of the health system.

### Mechanisms

#### Training and supervision

Mechanisms such as training and supervision ensure ANMs will become competent through quality pre-and in-service training and supportive supervision. Arrangements that influenced the practices of ANMs and equipped them with the knowledge and skills sets needed to accomplish the assigned tasks included clinical, communication (including counselling), organisational and administrative (including financial record keeping and computer) skills. In general, ANMs receive two types of training: pre-service training with a duration of 24 months (18 months theory plus six months practical) and in-service training. There had been frequent changes in the duration of ANM pre-service training.

In addition to pre-service training, ANMs require regular updates of knowledge and hands-on skills in the clinical areas of their work, for instance, assisting at childbirth. However, ANMs reportedly did not receive the required technical knowledge and skills **due to lack of hands-on experience** during their training. The following quote of an ANM who participated in an FGD illustrates the observation:

“*When they give Skilled Birth Attendance training*, *we have to go to the allotted hospital*. *Generally*, *these hospitals have interns and the junior staff there*. *So*, *we don’t get a chance to handle the actual case*. *We just observe everything*. *So*, *we do not get to do anything*. *If they keep this training at our place*, *it will be good*.”

Three ANMs who participated in the FGDs reported that they did not receive training on basic pharmacology for the medicines they were dispensing. Others were not comfortable in prescribing as they were not aware of the indications or contraindications for those medicines. However, the ANM pre-service training syllabus of the Indian Nursing Council includes common medicines used for emergencies and minor ailments, indications, dosage and actions [9,10]. These comments question the content of teaching and further suggest the need for continuous updates and refresher training on subjects such as commonly used medicines.

**The quality of ANM training** was affected by training institutions and approaches to trainings, availability of training materials (such as textbooks and curriculum) and training logistics (such as the duration and location of the training). Almost every ANM felt that practical, hands-on training should be included to improve results from the training. ANMs perceived that training with no practice session was not very useful, particularly if they were related to clinical skills. Whether **training institutions** were public or private institutions significantly influenced the quality of the ANMs trained. Due to unsatisfactory enforcement of the regulation of ANM training institutions, ANMs who graduated from private institutes which did not adhere to national guidelines were reported to be “sub-standard”. Some private institutions were reported to be missing required training aids. The length of training was considered insufficient to produce a qualified and competent ANM.

Furthermore, most ANMs did not receive regular refresher training primarily due to the absence of such opportunities or their inability to dedicate time for the same against other work priorities with limited human resources. Except for occasional monitoring visits to a purposeful sample of participants, in-service training institutions were **unable to conduct regular, structured evaluations after training due to lack of resources**.

In addition to training, there was a lack of **effective supervision** of ANMs at their workplaces. Health facility, district, and national interviewees unanimously perceived that the existing health system did not prioritise supervision as an essential element to improve the quality of the ANM programme. A national level government official commented:

“*It seems like there was no form of supportive supervision like on the job*. *Even if an LHV visited the ANM workplace*, *the main supervision she would do was on the report*, *on the register*, *on the data entry*. *When you ask ANMs about these supervisions*, *they will mention mainly on the reporting aspect but not on the technical*.”

The **existing supervisions focused on checking** if expected activities were carried out by looking at reports prepared by ANMs. Other important aspects of supervision such as problem-solving and clinical observation which could improve the quality of ANMs’ work were neglected. An ANM who participated in an FGD stated: “*If ANM is unable to perform any task supervisor should guide her or perform that task*. *But this does not happen in fact*.”

It was unclear who should supervise ANMs and how. In most cases, Lady Health Visitors (LHV) supervise ANMs. LHV is an experienced ANM who undergoes additional course of 6 months to qualify herself as a Lady Health Visitor. At times, health assistants (HA) and medical officers (MO) also undertook ANMs’ supervisions due to the lack of LHV positions in the facility. The HAs who participated in the interviews also commented that ANMs’ supervisions were considered part of their job within the PHC or sub-centres, but none of them could explain exactly what they were supposed to supervise. There was a unanimous opinion that **LHVs who once filled the role of an ANM should supervise ANMs**. Formal job descriptions for ANMs’ supervisors exist, but the role was implemented differently as there were differences among individual supervisors.

Almost every facility level participant (LHV and HA) who was required to supervise ANMs, reported the inability to conduct enough supervisory visits to ANMs, the main reason for which was “not having enough time”. Without enough supervisory visits, the LHV or HA had no way of knowing whether ANMs were providing quality services. Beyond the frequency of supervision, the content, quality, and purpose of supervision are instrumental in improving ANM’s performance. However, these differed across supervisors as there were **no objectively set guidelines and measures for supervisors**. Besides ANMs, interview participants from the facility, state, and national levels stated that ANMs’ interactions with their supervisors were limited mainly to reporting.

ANMs should be able to interact with their supervisors, as then they would learn from the interaction. Unfortunately, the current system does not support this and instead, it focuses on reporting the numbers of beneficiaries reached by the ANMs. Every ANM that participated in the study agreed that having opportunities to interact with supervisors motivated them for higher performance and fostered a good working environment. ANMs recalled their routine reporting to their supervisors, typically through verbal communication by telephone, in person or by text message whenever they had cases at health facilities or during home visits. In addition to supervisors’ visits to peripheral facilities (sub-centres), participants described ANMs’ visits to headquarters (PHCs) where ANMs retrospectively reported their activities as “supervisions”.

#### Accountability mechanisms

ANMs, MPWs, HAs and LHVs have specific job descriptions. However, ANMs were often the “responsible person-in-charge” at peripheral health facilities such as sub-centres. Coordination and collaboration among different health staff working in facilities were instrumental in creating a positive working environment conducive to greater productivity and efficiency. This was crucial particularly in resource-constrained situations when staffing levels for the provision of health service delivery were limited. In some facilities, ANMs were isolated without adequate support from other colleagues such as male MPWs while in other facilities, ANMs and male MPWs were working as a team. Key informants at the facility, state, and national level and ANMs stated that it would be helpful to receive support from male MPWs to provide good quality health service delivery to the beneficiaries.

The **male MPWs did not have formal responsibilities for work related to maternal and child health (MCH),** as MCH was not part of their written job description. Consequently, it was difficult to hold them accountable. In the absence of clear roles and responsibilities, especially for male MPWs, **ANMs felt that they were often held accountable unnecessarily**. ANMs felt they were being treated unfairly by their managers even when they have been covering the jobs of other health staff including male MPWs and HAs. ANMs were reportedly held accountable even at the block level meetings.

Many ANMs who participated in the study disclosed their discontent with the situation and the injustice of being held accountable while **others in the PHC were not held responsible**. ANMs described many challenges such as handling complicated childbirths in the absence of the medical officer responsible for the PHC. In addition to the lack of support at their workplaces, staff from referral facilities did not understand the challenges which ANMs faced as they were accustomed to placing blame on ANMs and ignoring their explanations. Some ANMs described situations when they made emergency referrals due to the severity of illness and the late arrival of patients. In such situations, ANMs could do nothing but just refer. Yet **ANMs received blame from referral facility staff for “not performing their duties”**.

It was a mixed picture in terms of community support. Some **ANMs perceived that communities at times blamed them** for not being responsible for the sub-centres. Consequently, ANMs were demoralised as they were not supported by their communities in their workplaces. At the same time, lack of support at work also led to the loss of trust from the communities because communities did not understand the challenges faced by ANMs. However, **women from the community who participated in the FGDs appreciated the work of ANMs** as they felt ANMs were “*friendly*” and like “*family members*” who “*spoke in a language the women understood*.” Unlike other staff in the health facilities, ANMs “*did not scold them when they went to a health facility and provided them mental support*”. Hence, “*their family allowed them to listen to the information given by ANM without any reservation*.” (Community FGD).

ANMs were held accountable for their duties and responsibilities, and there were some, though inadequate, formal performance monitoring mechanisms in the current health system. Importantly, the extent of the implementation of these mechanisms and the process of the appraisal was not clear. Some ANMs were not even aware of the existing rewards system in place or of the penalty for non-performance. ANMs highlighted that the weak appraisal of performance trapped their career progression. Most of their predecessors retired as ANMs without promotion. National level participants were aware that **ANMs do not have a career pathway to progress** apart from becoming an LHV closer to the age of retirement. Having an objective and structured system of performance appraisal would motivate ANMs “*to develop and love to work*.” The following quote from a national level participant exemplifies this observation:

*"They do not have any promotions*. *So*, *they do not have a career pathway*. *Once an ANM and she retire somewhere as LHV at the far end of their time*. *Now if you have such a system*, *who is going to work*? *So*, *at once the ANM joins*, *she should be able to know that in my 30 years’ career*, *I will be reaching maybe district PHN*, *I can become like that*. *I think that's everybody's [wish]*, *the system has to be conducive for that for people who are*, *performing well in their field*, *they should be given some incentive so that they look up to that*.*"* (KI-11)

In the absence of a robust formal system for performance accountability, **informal arrangements** emerged. ANMs appreciated any form of informal appreciation (words of thanks, certificates or recognition for their work) as this form of appreciation motivated them. Generally, the best ANM award from each district was selected based on the recommendations from the relevant block and PHC. However, not every ANM was aware of the process. Some ANMs posted at PHCs received the best ANM of the year award although many ANMs from sub-centres discovered the existence of such an award only during the focus group discussions. In some PHCs, the best ANM was nominated for her performance by the officials (medical officers, block and district health officers). ANMs from the sub-centres felt excluded as they were not aware of the performance awards at PHCs. Some ANMs were demotivated as they were “*always the last to receive any prize*.”

Despite the weakness in the informal performance appraisal system, the system provided scope for individual managers to **penalise non-performance or apply sanctions** against ANMs. Some ANMs perceived that they “*have not received any prize but only punishment*”, mostly in the form of a memo. A memo is issued by a relevant supervisor or manager of the ANM as part of a formal disciplinary action mechanism. In most cases, the medical officer or the individual in charge of the PHC issued a memo when an ANM failed to perform. An ANM was suspended from work after receiving memos more than three times. In some cases, **salaries or allowances of the ANM were reduced or cut** until ANM settled the complaint. While the performance of a health facility was a collective effort, ANMs felt that they received “*the total blame and everything was put on her*.*”*

## Discussion

Our study highlights that factors governing the performance of ANMs are crucial for this cadre to successfully contribute to achievement of universal health coverage. These factors generally govern not only the performance of ANMs but also that of the health system on a wider level. Using the Pawson and Tilley realist lens, we were able to identify “contextual factors” (regulatory system, infrastructure and resources, gender roles and norms and ANMs’ expanded scope of work) as well as “mechanisms” (accountability mechanisms, training and supervision) that influenced ANMs.

Ideally, standardised pre-service training should provide essential core skills and knowledge to become a qualified ANMs. Once ANMs are in service, they should receive regular, context-specific on-the-job training relevant to their tasks. For instance, the skills to handle and manage a high-risk pregnancy or complicated birth will be relevant for ANMs posted in a sub-centre in a remote, tribal village. However, this might not be relevant for ANMs posted at the PHC managed by a medical officer in an urban setting. Furthermore, the weakly enforced regulatory system governing ANM training undermines the potential of ANM cadre. Challenges with health system regulation are not unique to Maharashtra state as underlying governance issues influence the regulation of health systems in other states in India [18]. The consequences of gaps in regulating ANM training institutions are significant, leading to poor quality of training curriculum of health care providers who are inadequately trained to provide quality health services to the population. As ANMs are at the forefront of the primary healthcare system, it is essential to rectify these negative impacts and identify ways to mitigate this. According to Sheikh et al [18], there are three key regulatory functions: the presence of a law or policy; the presence of an independent organisation assigned to enact the prescribed law or policy; and the extent of the implementation. Our findings suggest that despite the availability of policy on paper, the actual implementation is questionable. Peters and Muraleedharan [19] have highlighted this issue concerning the regulatory system of the Indian health system. This is further compromised by resource constraints within the public systems such as logistical issues within the health system and delays within the judicial system [20]. Under such conditions, when professional associations are assigned to deliver regulatory control of their training institutions, they are reluctant to take actions against their own members due to conflict of interests, power differences and unclear roles and responsibilities [21]. Sheikh et al termed this situation as “regulatory capture”[21]. As a result, ill-qualified ANM training institutions continue to operate without the scrutiny of the regulatory system, highlighting gaps in the governance of the regulatory system in Pune district. The regulatory system should certainly scrutinise public as well as private ANM training institutions and ensure their compliance with inspection, accreditation, certification and production of qualified ANMs.

Gender roles and social norms within their society was another important social context that governed ANMs at their work place. As female health workers residing within the communities, ANMs faced different experiences without adequate safety measures at their workplaces. Similar findings have been reported in other studies: community-based frontline health workers had limitations due to gender roles and norms within their societies [22,23]. The consequences of gender roles demotivate ANMs because of their perceived lack of safety at their work places. Unfortunately, this finding was not unique to Pune as similar findings have been reported elsewhere [24,25].

The other important finding from this study is regarding accountability mechanisms governing ANMs in Pune district. Our study highlights the challenges in regard to internal accountability within the health system hierarchy and how it plays a crucial role in providing quality care due to the interactions among technical (ANM, LHV, HA and MO), managerial and political actors within the health system [26]. While the health system wants ANMs to be accountable for their responsibilities, formal responsibilities for another cadre such as male MPWs should also be clearly stated. Since 2016, preventive services of major non-communicable diseases (hypertension and diabetes) and three most common cancers (oral, cervix and breast cancers) have been added to the job descriptions of ANMs [27]. Furthermore, with the introduction of Ayushman Bharat scheme [11], ANMs will also be assigned at the Health and Wellness Centres (upgraded sub-centres) to provide a wide range of preventive, promotive, curative and rehabilitative services; however, they will not be in leadership positions as they are in their current roles in sub-centres. The roles and responsibilities of ANMs are subject to more changes under the new scheme. While the roles and responsibilities of ANMs have changed over time, it is equally important to reorient roles and responsibilities of other cadres from time to time, and to improve their accountability. The other bottleneck of the accountability mechanisms is the unclear implementation of formal appraisal and lack of career progression for ANM. Both affect motivation. Unfortunately, these are at the discretion of individual managers who can make use of their positions. George [28] reported a similar observation in Karnataka state, India, highlighting how people in power or with authority could make use of the disciplinary mechanisms and practised implicit “*quid-pro-quo*”.

The fifth factor governing the performance of ANMs at their workplaces was training and monitoring systems such as lack of quality supervision mechanisms and regular on-the-job mentoring and refresher training. George [28] reported similar observation, highlighting how ANM programme lacked meaningful supervision. There is an urgent need to improve the approach of the existing supervision as its primary focus has been on reporting. ANMs have limited training with hardly any opportunities for refresher training but are responsible for carrying out their duties. Supportive supervision plays a crucial aspect to improve the quality of their work and learning. Ideal supervision should take place in their workplace with immediate feedback to the ANMs to assist in maximising their performances [29]. Bosch-Capblanch and Garner proposed three types of supervision to improve the performance of a supervisee: problem identification and solving; examination of information against expected outputs; and observation of the clinical practice [29]. The current supervision approach which ANMs receive focuses on examining the information against expected outputs by checking their reports. There was minimal clinical support at their workplaces with very little problem identification and solving. Checklists are the most common feature of supervising ANMs in an authoritative approach, rather than problem-solving and feedback. Similar observations were reported in a systematic review of Bosch-Capblanch and Garner as characteristics of supervision in developing countries [29]. Our study participants commented on not having enough time for supervision. Without enough supervisory visits, there is no way of knowing the conditions of ANMs in their workplaces. Unfortunately, this finding is not unique. Kok et al [30] highlighted how adjustment should be made to improve supervision of community health workers in their comparative analysis in four sub-Saharan countries. This is also in line with the finding of a systematic review of primary healthcare supervision in developing countries [29].

Erchick et al [26] defined supervision as a complex process to create change which involves different actors within a health system, touching upon different areas of accountability. Therefore, quality supervision will require initiation from the supervisor to make supervisory visits happen [26]. Unfortunately, such initiatives were not mentioned or documented in this study, highlighting a need to strengthen the role of the supervisors.

The success of ANM programmes is contingent on more than technical inputs. Focusing on the functions of ANMs in isolation will not achieve an effective cadre relevant to the needs of its population. Therefore, the study has used the realist approach of Pawson and Tilley to identify “context” and “mechanisms” that influence ANMs within a wider health system. The health system context should be addressed to achieve expected programme outcomes of frontline health workers [18,24]. Complex health system reforms must be implemented together with technical inputs for ANMs to enable responsiveness to the context. Social structure and health system hierarchies challenge the operations of ANMs in their workplaces which when compounded by resource constraints, creates practical challenges for everyone working in the PHC. Opaque accountability mechanisms make ANMs and other cadres working in the primary health system difficult to be accountable in the workplaces. Some factors are beyond the immediate focus of ANMs but are institutional factors that govern the performance of ANMs in their workplaces. The major barriers identified to the successful implementation of ANM programmes should encourage system thinking by health system policy makers and managers.

### Strengths and limitations

Despite the efforts of the research team to triangulate the data collection methods (FGDs, KIIs, observations) and data sources, it is possible that participants gave ‘expected’ responses, were reluctant to respond fully or may not have reflected their own opinions. Despite the use of realist lens to present the results, this study is not a realist evaluation as the realist lens was not used from the outset of the study (i.e., in the inception phase). The authors chose to use two theoretical components of a realist lens in presenting the results as the findings fit well within the realist theoretical framework. The realist lens has proven evidence to identify factors influencing complex interventions by highlighting how “context” interacts with different “mechanisms” [14]. In other words, in order to bring realistic and sustainable changes to a health system, understanding a full account of “context” and influencing “mechanisms” are essential and instrumental [18,30,31]. For that, the realist lens provides a valuable tool to analyse and present results by unpacking different contexts and mechanisms.

ANMs may have been reluctant to openly express their opinions during FGDs and interviews even though the researchers were not associated with their facilities and gave assurances of confidentiality. Therefore, the research team used open-ended questions (where applicable) to minimise interviewer/facilitator bias. Some of the data and its meaning/implications may have been lost in translation even though all efforts were made to ensure data integrity. This was particularly relevant for the FGDs, which were conducted in the local language.

The findings from this study may have limited generalisability beyond its immediate study sites (Pune district and Maharashtra state) as it included a limited number of stakeholders (interviewed at a specific point in time). Nevertheless, the study aims to identify factors that govern the performance of ANMs in their workplaces; hence some findings may be recognisable in other states of India.

### Implications for scholarship and practice

Findings from this case study on ANMs calls for a further analysis of regulatory architecture focusing on enforcement of policies governing training and deploying ANMs, together with institutional strengthening for better regulation of the health system within Pune district. The study findings support standardising training contents and regular refresher sessions.

ANMs’ commitment to their job and their critical role within the current health system positively support the need for clearer job description and career progression. Additional tasks that ANMs are asked to carry out over time should be reviewed to avoid confusing ANMs and giving excuses for accountability. This lack of internal accountability within the health system hierarchy with little incentives and motivation for one’s performance such as an unclear career pathway should also be addressed.

There is an urgent need to strengthen the existing system of supervision, particularly a need to strengthen the role of supervisors in supporting ANMs at their workplaces. Supportive supervision should be based on clear, measurable and realistic indicators. Supervision should focus on aspects such as on-the-job mentoring, problem solving, providing feedback and clinical supervision in addition to administrative matters and verifying the reports. The supervisors must understand that they are accountable to their supervisees.

## Conclusion

ANMs have been instrumental in the delivery of primary health care in India. However, the training and supervision that this cadre receives are inadequate to address their current responsibilities and to take on additional responsibilities in the future. Despite the importance of this cadre, studies that investigate broader health system factors governing the performance of ANMs in their workplaces are limited. Focusing on the tasks of the ANMs with technical inputs alone is insufficient to achieve the full potential use of these health workers who can cater to evolving health needs in a changing context. Systematic efforts to tackle factors governing ANMs in their workplaces can only produce a useful cadre that can play an important role towards achieving universal health coverage in India.

## Supporting information

S1 Appendix Appendix 1. Characteristics of study participants.
(DOCX)

S2 Appendix Appendix 2. Themes and sub-themes.
(DOCX)

S1 Guide Guides for key informant interviews—English and Marathi. (PDF)

S2 Guide Guides for focus group discussions—English and Marathi. (PDF)

S3 Guide Guide for observations. (PDF)

## Abbreviations

ANM: Auxiliary Nurse Midwife
ASHA: Accredited Social Health Activist
CMND: Communicable, maternal and newborn disorders
FGD: Focus group discussion
FRCH: Foundation for Research in Community Health
HA: Health Assistant
KII: Key informant interview
LHV: Lady Health Visitor
LSTM: Liverpool School of Tropical Medicine
MCH: Maternal and Child Health
MO: Medical Officer
MPW: Multipurpose Worker
NHM: National Health Mission
NCD: Non-communicable disease
NRHM: National Rural Health Mission
PHC: Primary Health Centre
SC: Sub-centre
