# Supplementary material for: Factors governing the performance of Auxiliary Nurse Midwives in India: A study in Pune district
Source: PLoS One. 2019 Dec 27;14(12):e0226831. doi: 10.1371/journal.pone.0226831 (PMC6934276; doi:10.1371/journal.pone.0226831)
Supplement: S1 Appendix — Characteristics of study participants. (DOCX) [file pone.0226831.s001.docx]

**Appendix 1. Characteristics of study participants**

**Appendix 1.A. Characteristics of KII participants in each health system level**

| Health system level | Institutions | Positions |  |
| --- | --- | --- | --- |
| National, State level | Ministry of Health; Parastatal organisations | Senior directors and advisors | 4 |
| District level | District Health Department  District training institutions (public) | Health officer  Principal, MO in-charge, Training tutor | 1  3 |
| Block, health facility | Block level hospitals  Primary health care centre (PHC)  Sub-centre | Block medical officer | 1 |
|  |  | Medical Officer-in-charge  Health assistants (HA)  Lady health visitors (LHV) | 1  1  2 |
| Total |  |  | **13** |

**Appendix 1.B. Characteristics of ANMs who participated in the FGDs**

| **No. of ANM participants** | **Age range in years** | **Type of facility stationed** | **No. of years as ANMs (min-max)** | **Distance from home to workplace (min-max) (average)** | **The last training received**  **(min-max)** | **No. of children under 5 reportedly treated in last 3 months** | **No. of women reportedly seen in last 3 months** |
| --- | --- | --- | --- | --- | --- | --- | --- |
| 7 (17%) | 24-49 | PHC | 5-16 years | 0-30 km  (9 km) | Ongoing-2 years | 100-1120 | 150-597 |
| 23(56%) |  | Sub-centre | 1-29 years | 0-40 km  (11.5 km) | Ongoing-5 years | 68-3500 | 12-750 |
| 1 (2%) | 50 above | PHC | 18 years | 0 km | Ongoing | 940 | 250 |
| 10 (24%) |  | Sub-centre | 18-30 years | 0-15 km  (7.4 km) | 5 months-10 years | 10-2225 | 19-800 |

**Appendix 1.C. Characteristics of community women who participated in the FGDs**

| **Age group in years** | **Total participants** | **Level of education** | **No. of under 5 children per woman** | **Average travel time from home to the nearest health facility using their usual means of transport (min-max)** | **Number of times assistance received from an ANM within the past 3 months (min-max)** |
| --- | --- | --- | --- | --- | --- |
| 20-30 | 14 (56%) | Under 10th grade | 1 to 3 | 2-30 min | 1 to 3 |
| 23-27 | 9 (36%) | >10^th^ grade but below graduate level | 1 to 2 | 5-30 min | 1 to 3 |
| 24-26 | 2 (8%) | Graduate level | 1 | 3-30 min | 1 to 3 |
