# Supplementary material for: Factors governing the performance of Auxiliary Nurse Midwives in India: A study in Pune district
Source: PLoS One. 2019 Dec 27;14(12):e0226831. doi: 10.1371/journal.pone.0226831 (PMC6934276; doi:10.1371/journal.pone.0226831)
Supplement: S2 Appendix — Themes and sub-themes. (DOCX) [file pone.0226831.s002.docx]

**Appendix 2. Themes and subthemes**

**Theme: Context**

| **Sub-themes** | **Codes** |
| --- | --- |
| Regulatory system | - Lack of standardisation of training institutions (content & quality) - Lack of enforcement of regulations on training institutions |
| Infrastructure and resources | - Distance and lack of regular public transports - Mobile network coverage/communication problems - Electricity - Water and sanitation problems at health facilities |
| Gender roles and norms | - Harassment from the community - Lack of protection measures at work place |
| ANMs’ expanded scope of work | - Population levels/coverage - Recording - Online reporting - Reduced home visits - Technical areas to cover, Tasks |

**Theme: Mechanisms**

| **Sub-themes** | **Codes** |
| --- | --- |
| Training | - Pre-service training-duration, content, quality - Training providers-public vs private - Training materials (text books, curriculum) - Training approach (theory, classroom based, practical, hands-on) - In-service or refresher training - Regulation & standardisation of training - Evaluation of training |
| Supervision | - Prioritisation of supervision/ understanding the importance of supervision - Structure of and focus of supervision - Role of supervisors-Who should be ANM supervisors? - Supervisors-roles and responsibilities, expected behaviour - Mentoring versus supervision |
| Accountability mechanisms | - Role of other health staff (MPW, LHV, HA) - Expectations from ANMs - Community support and trust - Appraisal for performance - ANMs’ career pathway - Penalty for actions - Disciplinary measures |
