# Supplementary material for: Factors governing the performance of Auxiliary Nurse Midwives in India: A study in Pune district
Source: PLoS One. 2019 Dec 27;14(12):e0226831. doi: 10.1371/journal.pone.0226831 (PMC6934276; doi:10.1371/journal.pone.0226831)
Supplement: S1 Guide — (PDF) [file pone.0226831.s003.pdf]

INTERVIEW TOPIC GUIDE-NATIONAL/STATE LEVEL

I. CHARACTERISTICS OF THE PARTICIPANTS

Interviewers need to fill out the KII log before the start of interview

II. ROLE OF ANM

1. We would like to hear about ANMs – specifically we are interested in how ANMs have contributed to the MCH in India. To start with, can you describe the role of your organization in programmes for ANMs?
2. Do you think that ANMs could reach everyone within the communities? (Including those in the rural, remote, disadvantaged population, etc.) How?

III. IMPLEMENTATION

1. Please tell us about key mechanisms supporting the planning and implementation of ANMs at national/ state level and below (**coordination mechanism**)
   Prompt: give example of recruitment, training, supervision, supplies, reporting, service delivery etc. How they integrate them?
2. How **do central level stakeholders of the ANM programme coordinate** with state, district and health centre (vertical coordination)?
3. What would you recommend to improve the coordination mechanism?
4. Which information do you use for planning, recruitment, training, quantification of supplies supervision, reporting, service delivery for ANMs? Do you think is there a need to improve? Why and how?
5. Do you think that ANMs are **relevant to the current context** in India/your state?
   Prompt: Do people value ANMs' services? In future, would you invest more in ANMs or in other cadres?
6. How do you perceive the relevance of ANM services to the needs of the communities (Which services should be prioritized? Would you take any of the services out? Would you add any?)
7. Can you please share your opinion regarding whether the expected role and trainings provided to ANMs match, how?
8. Do you think that in future there will be **continued demand & utilization of ANMs**?
9. How best to **sustain the capacity** (of the ANMs) that has been created?
10. Do you have any recommendations tosustain the capacity of the ANMs that has been created?
11. Are you aware of any upcoming programme revisions or policies regarding ANMs? If so, what are they? How do you find them?

- **Ask if they would like to add further comments**
- **Bring the meeting to a close by summarizing the main points**
- **Do not forget to say thank you to the participants for their time and active participation.**

INTERVIEW TOPIC GUIDE-DISTRICT LEVEL

I. CHARACTERISTICS OF THE PARTICIPANTS

Interviewers need to fill out the KII log before the start of interview

II. ROLE OF ANM

1. We would like to hear about ANMs – specifically we are interested in how **ANMs have contributed to the MCH** in your district. To start with, can you describe the role of your organization in programmes for ANMs?

   आम्हाला ANMs विषयीची माहिती समजून घ्यायची आहे-विशेषकरून आम्हाला हे जाणून घ्यायचे आहे की भारताच्या माता आणि बाल आरोग्य कार्यक्रमात ANMs कशाप्रकारे योगदान देतात. मुलाखतीच्या सुरुवातीला आम्हाला सांगा की ANMs साठी असलेल्या कार्यक्रमांमध्ये तुम्ही काम करीत असलेले कार्यालय/ केंद्र काय भूमिका बजावते?

2. Do you think that ANMs could reach everyone within the communities? (Including those in the rural, remote, disadvantaged population, etc.) How?

   तुम्हाला असे वाटते का की आरोग्य सुविधा देण्यासाठी समाजातील सगळ्या घटकांपर्यंत ANMs पोहचू शकतात (ग्रामीण भाग, दुर्गम भाग, वंचित घटक इत्यादी.), कशाप्रकारे?

3. How do you find the quality of services provided by ANMs?
   ANMs द्वारे पुरवल्या जाणाऱ्या (आरोग्य) सेवांच्या दर्जाबद्दल तुम्ही काय सांगाल?

III. IMPLEMENTATION

1. Please tell us about key mechanisms supporting the planning and implementation of ANMs at district level and below (**coordination mechanism**)

   ANMs केडर संदर्भातील योजना आणि त्यांची अंमलबजावणी यासाठी केंद्र,राज्य किंवा स्थानिक पातळीवर समन्वय साधण्यासाठी कशा प्रकारच्या यंत्रणा आहेत. त्यातील महत्वाच्या यंत्रणांविषयी सांगाल का?
   Prompt: give example of recruitment, training, supervision, supplies, reporting, service delivery etc. How they integrate them?

   जागा भरती, प्रशिक्षण, देखरेख, औषध आणि इतर साहित्य पुरवठा, वृतांतलेखन (reporting), विविध सेवा इत्यादी सर्व गोष्टींचा एकत्रित विचार केला जातो का? कसा?

2. How do district level stakeholders involved in the ANM programme coordinate with national level and with sub-district, health centre and village (vertical coordination)? Please provide practical examples.

   ANM कार्यक्रमासाठी जबाबदार असलेले जिल्हा पातळीवरचे अधिकारी केंद्रीय, राज्य आणि तालुका, तसेच आरोग्य केंद्रे आणि गावे ह्यांच्या बरोबर कशा पद्धतीने समन्वय साधतात? ह्या बाबत काही उदाहरणे द्याल का?

3. What do you recommend to improve the coordination mechanism(s)?

   समन्वय यंत्रणा अधिक प्रभावी होण्यासाठी तुमच्या काही शिफारसी/ सूचना आहेत का?

4. Which information do you use for planning, recruitment, training, quantification of supplies supervision, reporting, service delivery etc. for ANMs?

   ANMs केडर संबधित योजना ठरवण्यासाठी तसेच जागांची भरती, प्रशिक्षण, देखरेख, औषध आणि इतर साहित्य पुरवठा, वृतांतलेखन (reporting), विविध सेवा इत्यादी गोष्टींचे नियोजन करण्यासाठी तुम्ही कुठल्या माहितीचा वापर करता?

5. Do you think that ANMs are **relevant to the current context** in your district?

   तुमच्या जिल्ह्याच्या सद्यस्थितीचा विचार करता ANMs कितपत उपयुक्त/ गरजेच्या आहेत असे तुम्हाला वाटते?

6. Prompt: Does it address people's priorities? Do people value ANMs' services? In future, would you invest more in ANMs or in other cadres?

   ANMs द्वारे दिल्या जाणाऱ्या सेवा ह्या लोकांच्या गरजांना अनुसरून का? ANMs द्वारे दिल्या जाणाऱ्या सेवा लोकांचा दृष्टीने महत्वाच्या आहेत का? भविष्यात जर कुठल्या केडर मध्येगुंतवणूक करायची असेल तर कुठल्या केडरमध्ये कराल?

7. How do you perceive the relevance of ANM services to the needs of the communities (Which services should be prioritized? Would you take any of the services out? Would you add any?

   तुमच्या मते ANMs द्वारे दिल्या जाणाऱ्या सेवा ह्या लोकांच्या गरजांना अनुरूप आहेत का? कुठल्या सेवांना प्राधान्य दिले पाहिजे? तुम्हाला ह्यापैकी कुठल्या सेवा कमी केल्या जाव्यात असे वाटते का? किंवा कुठल्या प्रकारच्या सेवांचा समावेश केला जावा असे वाटते का?

8. Can you please share your opinion regarding whether the expected role and trainings provided to ANMs match, how?

   ANMs ची अपेक्षित भूमिका आणि त्यांना मिळणारे प्रशिक्षण हे एकमेकांना पूरक आहेत असे तुम्हाला वाटते का? का?

9. Do you think that in future there will be **continued demand & utilization of ANMs**? Why?

   भविष्यात ANMsद्वारे दिल्या जाणाऱ्या सेवांना सतत मागणी असेल तसेच त्या सेवांचा वापर केला जाईल असे तुम्हाला वाटते का? का?

10. How best to sustain the capacity (of the ANMs) that has been created?

    ANMs मध्ये निर्माण झालेल्या क्षमता कशाप्रकारे कायमस्वरूपी टिकवून ठेवता येतील?

11. Do you have any recommendations to sustain the capacity of the ANMs that has been created?

ANMs मध्ये निर्माण झालेल्या क्षमता कशाप्रकारे कायमस्वरूपी टिकवून ठेवण्यासाठी तुमच्या काही शिफारसी/सूचना आहेत का?

12. Are you aware of any upcoming programme revisions or policies regarding ANMs? If so, what are they? How do you find them?

    ANMs संबंधित कार्यक्रमात केल्या जाणाऱ्या नवीन तरतुदी, नवीन धोरणे ह्याबद्दल तुम्हाला काही माहिती आहे का? ह्या बद्दल तुम्हाला काय वाटते?

- **Ask if they would like to add further comments**
- **Bring the meeting to a close by summarizing the main points**
- **Do not forget to say thank you to the participants for their time and active participation.**

INTERVIEW TOPIC GUIDE-ANM TRAINING INSTITUTIONS

I. CHARACTERISTICS OF THE PARTICIPANTS

Interviewers need to fill out the KII log before the start of interview

II. ROLE OF ANM

1. We would like to hear about ANMs – specifically we are interested in how ANMs have contributed to the MCH in your district. To start with, can you describe the role of your organization in programmes for ANMs?

   आम्हाला ANMs विषयीची माहिती समजून घ्यायची आहे-विशेषकरून आम्हाला हे जाणून घ्यायचे आहे की भारताच्या माता आणि बाल आरोग्य कार्यक्रमात ANMs कशाप्रकारे योगदान देतात. मुलाखतीच्या सुरुवातीला आम्हाला सांगा की ANMs साठी असलेल्या कार्यक्रमांमध्ये तुम्ही काम करीत असलेले कार्यालय/ केंद्र काय भूमिका बजावते?

2. Do you think that ANMs could reach everyone within the communities? (Including those in the rural, remote, disadvantaged population, etc.) How?

   तुम्हाला असे वाटते का की आरोग्य सुविधा देण्यासाठी समाजातील सगळ्या घटकांपर्यंत ANMs पोहचू शकतात (ग्रामीण भाग, दुर्गम भाग, वंचित घटक इत्यादी.), कशाप्रकारे?

3. How do you find the quality of services provided by ANMs?

   ANMs द्वारे पुरवल्या जाणाऱ्या (आरोग्य) सेवांच्या दर्जाबद्दल तुम्ही काय सांगाल?

III. OPINION AND SUGGESTION ABOUT ANM TRAINING

1. Can you please share us information regarding ANM training, especially we would like to know how is the syllabus designed? When it was first established? Was it revised since then?

   तुम्ही आम्हाला ANM प्रशिक्षण कार्यक्रमाबद्दल माहिती दयाल का? खास करून आम्हाला हे सांगा की प्रशिक्षण अभ्यासक्रम कसा तयार केला जातो? सर्वप्रथम हा प्रशिक्षण अभ्यासक्रम कधी तयार केला गेला? तसेच ह्या अभ्यासक्रमात बदल केले गेले का? केव्हा?

2. Particular inquiry about in-service training material: What is your experience regarding making the in-service training material locally relevant (adaptations, translation etc.)?

   सेवेत रुजू झाल्यावर (असताना) घेतल्या जाणाऱ्या प्रशिक्षण कार्यक्रमांबद्दल माहिती: सेवेत असताना जे प्रशिक्षण कार्यक्रम असतात त्यासाठी प्रशिक्षण साहित्य बनवताना ते स्थानिक पातळीवर योग्य ठरेल यासाठी काय केले जाते? (बदल, भाषांतर इत्यादी.)

3. How do you find pre-service syllabus for ANMs- particularly with regards to content, coverage, teaching methods, tools, duration, need for innovation or any other approach, etc.?

   ANM प्रशिक्षण अभ्यासक्रम विषयी तुमची काय मते आहेत- विषय (घटक), विषयांचा अंतर्भाव, प्रशिक्षण पद्धती, प्रशिक्षण साधने, प्रशिक्षण कालावधी, नाविन्यपूर्ण पद्धतींची आवश्यकता, इत्यादी?

4. Do you think this training provided caters to ANMs' job profile? Does it cover any field based responsibilities when ANMs are in service?

   तुमच्या मते दिले ANMs ना दिले जाणारे प्रशिक्षण हे ANMsना करायला लागणाऱ्या कामांच्या दृष्टीने योग्य/ परिपूर्ण आहे का?ह्या प्रशिक्षणामध्ये ANMs ना गावपातळीवर कराव्या लागणाऱ्या कामांचा समावेश असतो का?

5. Do ANMs in-service receive any refresher trainings? If so, how often do they receive?

   सेवेत रुजू असणाऱ्या ANMs साठी काही पुनःप्रशिक्षण कार्यक्रम असतात का? जर हो तर कधी (किती दिवसांनी) असतात?

6. How do you find the current venue and method of training (classroom/ field based/ simulation/ interactive/ tablet based/ team training etc.; duration of training)?

   सध्या दिल्या जाणाऱ्या प्रशिक्षणासाठीचे ठिकाण आणि प्रशिक्षण पद्धती विषयी तुम्हाला काय वाटते?(वर्ग आणि व्याख्यान/ कामाच्या ठिकाणी/ simulation (प्रात्यक्षिक)/ tablet computer वापरून/ गट प्रशिक्षण इत्यादी. प्रशिक्षण कालावधी इत्यादी.)

7. Any views on preferred venue, method of training?

   अधिक सोयीचे ठिकाण, प्रशिक्षणाची पद्धत ह्याबद्दल तुमच्या काही सूचना आहेत का?

8. What are the challenges related to training at central place?

   मध्यवर्ती ठिकाणी प्रशिक्षण कार्यक्रम घेण्यामध्ये कुठली कुठली आव्हाने आहेत?
   Prompt: Pune vs decentralised i.e at various block levels

   (पुणे किंवा तालुक्याची ठिकाणे तुलनात्मक चित्र)

- **Ask if they would like to add further comments**
- **Bring the meeting to a close by summarizing the main points**
- **Do not forget to say thank you to the participants for their time and active participation.**

INTERVIEW TOPIC GUIDE-ANM ASSOCIATION

I. CHARACTERISTICS OF THE PARTICIPANTS

Interviewers need to fill out the KII log before the start of interview

II. ROLE OF PARTICIPANTS IN ANM PROGRAMME

1. We would like to hear about ANMs – specifically we are interested in how ANMs have contributed to the MCHin your district. To start with, can you describe the role of your organization in programmes for ANMs?

   आम्हाला ANMs विषयीची माहिती समजून घ्यायची आहे-विशेषकरून आम्हाला हे जाणून घ्यायचे आहे की भारताच्या माता आणि बाल आरोग्य कार्यक्रमात ANMs कशाप्रकारे योगदान देतात. मुलाखतीच्या सुरुवातीला आम्हाला सांगा की ANMs साठी असलेल्या कार्यक्रमांमध्ये तुम्ही काम करीत असलेले कार्यालय/ केंद्र काय भूमिका बजावते?

2. Do you think that ANMs could reach everyone within the communities? (Including those in the rural, remote, disadvantaged population, etc.) How?

   तुम्हाला असे वाटते का की आरोग्य सुविधा देण्यासाठी समाजातील सगळ्या घटकांपर्यंत ANMs पोहचू शकतात (ग्रामीण भाग, दुर्गम भाग, वंचित घटक इत्यादी.), कशाप्रकारे?

3. How do you find the quality of services provided by ANMs?

   ANMs द्वारे पुरवल्या जाणाऱ्या (आरोग्य) सेवांच्या दर्जाबद्दल तुम्ही काय सांगाल?

4. What is your opinion about training of ANMs with regard to priority subjects, approach of training, tools, venue?

   ANM प्रशिक्षण कार्यक्रमाबद्दल तुमची काय मते आहेत? (प्राधन्यक्रमाचे विषय, प्रशिक्षण पद्धती, प्रशिक्षण साधने, प्रशिक्षण स्थळ(ठिकाण) इत्यादी.

5. We would like hear your views on who should be the trainers, assessment of training etc.

   प्रशिक्षक कोण असावेत, प्रशिक्षणाचे मूल्यमापन कसे करावे याबद्दल तुमची मते आम्हाला सांगा.

6. How can we inculcate self-learning among the ANMs? What ideas/ suggestion do you have?

   ANMs मध्ये स्वयंशिक्षणाची (सेल्फ-लर्निंग) वृत्ती वाढावी ह्यासाठी काय करता येऊ शकेल? ह्याविषयी तुमच्या काय सूचना आहेत?

   Prompt: Explain the activities we intend to undertake in the project- capacity building workshops for supervisors, ANM exchange visit, ANMs to attend conference, arranging skills mall.

(ह्या प्रकाल्पामध्ये आम्ही, ANM निरीक्षकांसाठी क्षमता वृद्धी कार्यशाळा, इतर ठिकाणची कार्यपद्धती बघण्यासाठी ANMs भेटी, राष्ट्रीय/ आंतरराष्ट्रीय कॉन्फरन्सेस मध्ये ANMs चा सहभाग, स्किल्स मॉल्स इत्यादी कार्यक्रम आयोजित करणार आहेत)

Probe: ask the respondent to share her views on the know-hows- how should such an activity be arranged? How can the ANMs be involved in this activity?

7. How do you find the involvement of public health sector in such activity? How can we improve this? How best can we sustain such activity?

   ह्या प्रकारच्या कार्यक्रमांमध्ये सामाजिक आरोग्य विभागाची गुंतवणूक/ भूमिका/ योगदान काय असते? ह्या मध्ये आपण कशा प्रकारे सुधारणा करू शकतो? अशा प्रकारचे कार्यक्रम कायम चालू रहावेत यासाठी काय काय केले पाहिजे?

8. How can ANM association help/ facilitate FRCH to undertake these activities?

   FRCH द्वारे घेतल्या जाणाऱ्या ANM कौशल्य विकास/ वृद्धी कार्यक्रमांमध्ये ANM असोसिएशनची कशाप्रकारे मदत होऊ शकेल?

9. Do you think that in future there will be **continued demand & utilization of ANMs**?Why?

   भविष्यात ANMs द्वारे दिल्या जाणाऱ्या सेवांना सतत मागणी असेल तसेच त्या सेवांचा वापर केला जाईल असे तुम्हाला वाटते का? का?

10. How best to sustain the capacity (of the ANMs) that has been created?

    ANMs मध्ये निर्माण झालेल्या क्षमता कशाप्रकारे कायमस्वरूपी टिकवून ठेवता येतील?

11. Do you have any recommendations to sustain the capacity of the ANMs that has been created?

    ANMs मध्ये निर्माण झालेल्या क्षमता कशाप्रकारे कायमस्वरूपी टिकवून ठेवण्यासाठी तुमच्या काही शिफारसी/सूचना आहेत का?

12. Are you aware of any upcoming programme revisions or policies regarding ANMs? If so, what are they? How do you find them?

    ANMs संबंधित कार्यक्रमात केल्या जाणाऱ्या नवीन तरतुदी, नवीन धोरणे ह्याबद्दल तुम्हाला काही माहिती आहे का? ह्या बद्दल तुम्हाला काय वाटते?

- **Ask if they would like to add further comments**
- **Bring the meeting to a close by summarizing the main points**
- **Do not forget to say thank you to the participants for their time and active participation.**

INTERVIEW TOPIC GUIDE-ANM SUPERVISORS

I. CHARACTERISTICS OF THE PARTICIPANTS

Interviewers need to fill out the KII log before the start of interview.

II. SCOPE OF WORK

1. Based on your experience working as an **ANM supervisor** which **tasks** are you expected to carry out?

ANMपर्यवेक्षक म्हणून काम करताना असलेल्या अनुभवांवरून ANM निरीक्षकाने काय कामे करणे अपेक्षित असते?

2. Where do you conduct the supervision? (In the health facility? In the community?)

ANM पर्यवेक्षक म्हणून ANMs च्या कुठल्या कामाचे पर्यवेक्षण करता? (ANMआरोग्य केंद्रात करत असलेल्या कामांचे/ ANM गावपातळीवर करत असलेल्या कामांचे)

3. What activities do you generally supervise? How? How frequently? Any structure/ tool for supervision?

साधारणपणे कुठल्या कुठल्या गोष्टींचेपर्यवेक्षण केले जाते? किती काळाने? पर्यवेक्षणासाठी काही आराखडा/साधने आहे का?

4. What happens to the information you collect during supervision? (feedback, reporting, analysis, revisions etc.)

ANMs च्या कामाच्या देखरेखीदरम्यान जी माहिती गोळा केली जाते त्या माहितीचे पुढे काय होते? (feedback, reporting, analysis, revisions etc.)Can you please share your challenges in conducting these activities?)

> ***Probe***: If the supervisors describe health system challenges such as shortage of staff or transport problem, ask them how have they managed to overcome those challenges? Try to encourage them to narrate specific case stories rather than general comments or statements.

III. OPINION AND SUGGESTION ABOUT ANM SUPERVISION

1. Who is the best person (designation wise) to supervise the work the ANMs undertake?

ANMs च्या कामावर देखरेख ठेवण्याच्या दृष्टीने कोणती व्यक्ती अगदी योग्य आहे असे तुम्हाला वाटते?

2. How should such supervision take place?

अशी देखरेख कशा प्रकारे ठेवता येईल?

IV. ANM'S KNOWLEDGE, ATTITUDE AND PRACTICES:

1. What do you think about the **level of ANMs' knowledge and skills** in your catchment area? (For instance, as it relates to ANC, delivery, PNC, family planning, child health)

तुमच्या भागातील ANMs चे त्यांच्या विषयाचे ज्ञान आणि कौशल्य ह्याविषयी तुमचे काय म्हणणे आहे? (उदा:ANC, delivery,PNC, family planning, child health etc.)

2. In what areas do you think that ANMs need to improve their knowledge, and skills to provide quality services? Why?

असे कुठले विषय/ गोष्टी आहेत का, ज्या मध्ये ANMs नी त्यांचे ज्ञान आणि कौशल्य वाढविल्यास त्या अधिक दर्जेदार सेवा देऊ शकतील आहे असे तुम्हाला वाटते?

3. How do you find **ANMs' adherence to the protocols and guidelines** in your catchment area within the last one year? (For instance, ANC, delivery, PNC, family planning, child health)

गेल्या एक वर्षाचा विचार करता तुमच्या भागातील ANMs त्यांची कामे, दिलेल्या प्रोटोकॉल्स आणि गाईडलाईन्स प्रमाणे करतात का?

4. How do you find the **community access to ANMs** within this district within the last one year?

गेल्या एक वर्षाचा विचार करता तुमच्या भागातील लोकांसाठी ANMs द्वारे दिलेल्या सेवा मिळवणे/ सेवांपर्यंत पोहचणे कितपत शक्य आहे?

5. In which areas of ANM services would you like to improve? Why?

ANMsद्वारे दिल्या जाणाऱ्या सेवांपैकी कुठल्या सेवांमध्ये सुधारणा व्हावी असे तुम्हाल वाटते? का?

6. How do you find the **ANMs' attitude** in service provision in your facility?

तुमच्या भागातील ANMs चा सेवा देताना कसा दृष्टीकोन असतो असे तुम्हाला वाटते?

7. In what areas do you think that ANMs should improve? Why?

तुमच्या मते ANMs मध्ये कुठल्या बाबतीत सुधारणा व्हायला पाहिजे? का?

V. BARRIERS AND FACILITATORS TO SERVICES:

1. Which factors **facilitate** ANMs to accomplish their tasks successfully?

असे कुठले घटक आहेत की जे ANMs त्यांची कामे यशस्वी होण्यासाठी मदत करतात.

2. What are the **barriers** for ANMs to accomplish their tasks?

ANMs च्या कामात अडथळे आणणारे घटक कोणते?

Probe: If the supervisors describe health system challenges such as shortage of staff or medical supplies, ask them how have they managed to overcome those challenges? Try to encourage them to narrate specific case stories rather than general comments or statements.

- **Ask if they would like to add further comments**
- **Bring the meeting to a close by summarizing the main points**
- **Do not forget to say thank you to the participants for their time and active participation.**
