# Supplementary material for: Factors governing the performance of Auxiliary Nurse Midwives in India: A study in Pune district
Source: PLoS One. 2019 Dec 27;14(12):e0226831. doi: 10.1371/journal.pone.0226831 (PMC6934276; doi:10.1371/journal.pone.0226831)
Supplement: S2 Guide — (PDF) [file pone.0226831.s004.pdf]

FOCUS GROUP DISCUSSION GUIDE-ANM

I. CHARACTERISTICS OF THE PARTICIPANTS

Facilitators need to fill out the FGD log before the start of the FGD.

II. SCOPE OF WORK

Based on your experience working as an ANM, which tasks are you expected to accomplish?

Prompt: Do you do any of the following: ANC, child birth, PNC, family planning, child health, other primary health care?

Probe: Which tasks do you actually carry out in reality and why? How much of your time do you spend on MNH as compared to other tasks?

ANM म्हणून आजवरच्या अनुभवात तुम्ही कोणकोणती कामे करणे अपेक्षित आहे?

आधाराचे मुद्दे: पुढीलपैकी कोणकोणती कामे तुम्ही करता-ANC, प्रसूती, PNC, कुटुंब नियोजन, मुलांच्या तब्येतीची काळजी, अन्य प्राथमिक आरोग्य सेवा.

चौकशी करा-तुम्ही प्रत्यक्षात कोणती कामे करता आणि का? इतर कामांच्या तुलनेत MNH म्हणून करण्याच्या कामांवर तुम्ही किती वेळ खर्च करता?

III. ANM'S TRAINING, SUPERVISION:

1) Can you please share us about training that you have received so far to work as an ANM?
   Prompt: Clinical skills, managerial skills including record keeping or communication skills.
   ANM म्हणून काम करण्यासाठी तुम्हाला आजवर मिळालेल्या प्रशिक्षणासंदर्भात कृपया आम्हाला माहिती देऊ शकाल का?
   आधाराचे मुद्दे: वैद्यकीय कौशल्ये, रेकॉर्ड ठेवणे किंवा संवाद कौशल्ये यांसह व्यवस्थापकीय कौशल्ये

2) Do you have any training in mind that you think can improve your knowledge and skills?
   Prompt: topics, approach, use of innovative tools, venue (block/district level), duration etc.
   तुमचे ज्ञान आणि कौशल्ये वाढू शकतील, असे एखादे प्रशिक्षण तुमच्या मनात आहे का?
   आधाराचे मुद्दे: विषय, दृष्टीकोन, अभिनव साधनांचा वापर, ठिकाण(गट/जिल्हा पातळी), कालावधी

3) Please tell us about the preferred place and preferred duration of such training.
   अशा प्रशिक्षणासाठी तुम्हाला कोणते ठिकाण आणि कोणता कालावधी अधिक योग्य वाटतो, हे आम्हाला सांगा.

4) After attending any such training, how would one really understand and measure whether that training was useful and could make any impact on the knowledge, skills that you have and ultimately on the services you deliver?

असे प्रशिक्षण पूर्ण केल्यावर, ते उपयुक्त होते अथवा नाही आणि तुमच्या ज्ञान आणि कौशल्यावर, तसेच अंतिमतः तुम्ही देत असलेल्या सेवांवर त्याचा काय प्रभाव पडू शकेल, हे एखाद्याला कसे समजेल आणि त्याचे मोजमाप कसे करता येईल?

Probe: Discuss - why against the probes- no previous training on that subject/ newly emerged subject/ never covered in the syllabus/ difficult to understand/ no demonstration received only theoretical training so far etc.

चौकशी करा- चर्चा: चौकशीच्या विरुद्ध का-त्या विषयावर आधीचे प्रशिक्षण झालेले नसणे/नव्याने उदयाला आलेला विषय/ अभ्यासक्रमात समावेश नसलेला विषय/समजायला अवघड/ आजपर्यंत या विषयावर फक्त सैद्धांतिक प्रशिक्षण मिळालेले असणे, प्रात्यक्षिकांचा अनुभव नसणे इ.

5) Are there protocols and guidelines that you need to follow as an ANM? What are they?
Prompt: Protocols and guidelines on ANC, child birth, PNC, family planning, child health, other primary health care. Are they readily available to you?

ANM म्हणून तुम्हाला काही शिष्टाचार, मार्गदर्शक सूत्रे पाळायला लागतात का? ती काय आहेत?
आधाराचे मुद्दे: ANC, प्रसूती, PNC, कुटुंब नियोजन, मुलांचे आरोग्य, अन्य प्राथमिक आरोग्यसेवा, या विषयीचे शिष्टाचार आणि मार्गदर्शक सूत्रे. हे तुम्हाला सहज उपलब्ध आहे का?

6) How do you find them? Do you find them useful or not? Why?

तुम्हाला ही सूत्रे/शिष्टाचार कसे वाटतात? तुम्हाला ते उपयुक्त वाटतात का नाही? का?

7) How do you find you and other ANMs' adherence to the protocols and guidelines in your area within the last one year? Are there barriers preventing you from adhering to these protocols and guidelines?

गेल्या वर्षभरात तुम्ही आणि इतर ANMsनी या शिष्टाचारांचा आणि मार्गदर्शक सूत्रांचा किती अवलंब केला आहे, असे तुम्हाला वाटते? या शिष्टाचारांचा आणि मार्गदर्शक सूत्रांचा अवलंब करण्यासाठी तुम्हाला रोखणारे काही अडथळे आहेत का?

8) We would like to know how do you coordinate with your trainers/ supervisors for your work? How often? Did they come to see you?

तुमच्या कामासाठी तुम्ही तुमच्या प्रशिक्षकांशी/पर्यवेक्षकांशी कसा समन्वय साधता? किती वेळा? ते तुम्हाला भेटायला आले होते का?

9) Did they visit while you were doing some activities (home visits of pregnant and postnatal women, delivery, family planning services, etc.)?

तुम्ही प्रत्यक्ष काम करत असताना(गर्भवती आणि प्रसूत झालेल्या स्त्रीयांना त्यांच्या घरी जाऊन दिलेल्या भेटी, प्रसूती, कुटुंब नियोजन सेवा, इ.) त्यांनी भेट दिली का?

10) How do you find that process of supervision? Anything that you want to change?

ती पर्यवेक्षणाची प्रक्रिया तुम्हाला कशी वाटते? तुम्हाला त्यात काही बदल करावासा वाटतो का?

11) How is your work generally assessed? Are there any rewards/ penalty based on the assessment?

तुमच्या कामाचे मूल्यमापन साधारणतः कसे केले जाते? या मूल्यमापनावर आधारित काही बक्षिसे/दंड असतो का?

12) Please tell us about the topics you want to get trained / retrained in. (Try to arrive at a list, and involve the group to prioritize the topics)

कोणत्या विषयांवर तुम्हाला प्रशिक्षण/ पुनर्प्रशिक्षण हवे आहे, ते कृपया सांगा. (विषयांची यादी तयार करायचा प्रयत्न करा आणि विषयांचे प्राधान्य ठरविण्यासाठी उद्‌दिष्ट गटाला सहभागी करून घ्या).

13) You have given priority to (list of topics) all these topics. Let us take each of the topic and discuss.

या सगळ्या विषयांना (यादीतील विषय) तुम्ही प्राधान्य दिले आहे. आता प्रत्येक विषय घेऊन त्यावर चर्चा करूया.

IV. BARRIERS AND FACILITATORS TO SERVICES:

1) Which factors facilitate you to accomplish your duties and tasks as an ANM successfully?

ANM म्हणून तुमच्या जबाबदाऱ्या आणि कामे यशस्वीपणे पूर्ण करण्यासाठी कोणत्या घटकांची मदत होते?

2) What are the barriers to accomplish your duties and tasks?

तुमच्या जबाबदाऱ्या आणि कामे पूर्ण करताना कोणते अडथळे येतात?

Probe:If ANMs describe health system challenges such as shortage of staff or medical supplies, ask them how have they managed to overcome those challenges? Try to ask them specific case stories/ examples rather than general comments or statements.

चौकशी करा:जर मनुष्यबळाची (स्टाफची) किंवा वैद्‌यकीय पुरवठ्याची कमतरता यासारख्या आव्हानांचे ANMs नी वर्णन केले तर या आव्हानांवर त्यांनी कशा प्रकारे मात केली आहे, हे त्यांना विचारा. ढोबळ विधाने किंवा प्रतिक्रियांऐवजी विशिष्ट उदाहरणे/ केसेस विचारून घेण्याचा प्रयत्न करा.

- **Ask if they would like to add further comments**
- **Bring the meeting to a close by summarizing the main points**
- **Do not forget to say thank you to the participants for their time and active participation.**

FOCUS GROUP DISCUSSION GUIDE-SKILLS LAB TRAINED ANMS

I. CHARACTERISTICS OF THE PARTICIPANTS

1) Facilitators need to fill out the FGD log before the start of the FGD.

II. SKILLS LAB TRAINING

1. Can you please share us about skills lab training that you have received? What subjects are taught during this training? (Try to seek as much details as possible)

   तुम्हाला मिळालेल्या कौशल्य प्रयोगशाळेतील प्रशिक्षणाविषयी आम्हाला सांगू शकाल का? या प्रशिक्षणादरम्यान तुम्हाला कोणते विषय शिकवले जातात? (जास्तीत जास्त तपशील मिळवायचा प्रयत्न करा).

2. What is your experience so far on skills lab training? (spontaneous responses)
3. तुम्हाला मिळालेल्या कौशल्य प्रयोगशाळेतील प्रशिक्षणाविषयी एकंदर अनुभव कसा आहे?

   Probe: Was it different from other trainings you received? If yes, how? (mode of training, trainers, content, assessment, supervision etc.)

   चौकशी करा:तुम्हाला आजवर मिळालेल्या प्रशिक्षणांपेक्षा हे प्रशिक्षण वेगळे होते का? जर असेल, तर कसे?(प्रशिक्षण पद्धती, प्रशिक्षक, आशय, मूल्यमापन, पर्यवेक्षण इ.)

4. Do you think this training will make any difference to your routine work once you go back? Can you please explain?

   तुम्ही परत नेहमीचे काम सुरु कराल तेव्हा या प्रशिक्षणामुळे त्यात काही फरक पडेल, असे तुम्हाला वाटते का?

5. How can such skill lab training be improved?

   कौशल्य प्रयोगशाळा प्रशिक्षणात सुधारणा कशी करता येऊ शकते?

6. Please tell us about the topics you want to get trained / retrained in. (Try to arrive at a list, and involve the group to prioritize the topics)

   तुम्हाला ज्या विषयांचे प्रशिक्षण/पुनर्प्रशिक्षण हवे आहे, ते विषय सांगा. (विषयांची यादी तयार करायचा प्रयत्न करा आणि विषयांचे प्राधान्य ठरविण्यासाठी उद्दिष्ट गटाला सहभागी करून घ्या).

7. You have given priority to (list of topics) all these topics. Let us take each of the topic and discuss.

   या सगळ्या विषयांना (यादीतील विषय) तुम्ही प्राधान्य दिले आहे. आता प्रत्येक विषय घेऊन त्यावर चर्चा करूया.

   Probe: Discuss - why against the probes- no previous training on that subject/ newly emerged subject/ never covered in the syllabus/ difficult to understand/ no demonstration received only theoretical training so far etc.

   चौकशी करा: चर्चा-चौकशीच्या विरुद्ध का - त्या विषयावर आधीचे प्रशिक्षण झालेले नसणे/नव्याने उदयाला आलेला विषय/ अभ्यासक्रमात समावेश नसलेला विषय/समजायला अवघड/ आजपर्यंत या विषयावर फक्त सैद्धांतिक प्रशिक्षण मिळालेले असणे, प्रात्यक्षिकांचा अनुभव नसणे इ.

8. Please tell us about the preferred place and preferred duration of such training.

   अशा प्रशिक्षणासाठी तुम्हाला कोणते ठिकाण आणि कोणता कालावधी अधिक योग्य वाटतो, हे आम्हाला सांगा.

9. After attending any such training, how would one really understand and measure whether that training was useful and could make any impact on the knowledge, skills that you have and ultimately on the services you deliver?

   असे प्रशिक्षण पूर्ण केल्यावर, ते उपयुक्त होते अथवा नाही आणि तुमच्या ज्ञान आणि कौशल्यावर, तसेच अंतिमतः तुम्ही देत असलेल्या सेवांवर त्याचा काय प्रभाव पडू शकेल, हे एखाद्याला कसे समजेल आणि त्याचे मोजमाप कसे करता येईल?

   Probe: What are your ideas/ what can be the strategies? Who would implement these ideas/ strategies? How (details for implementation of the specifically mentioned strategy)? (For seeking their views about after training monitoring, supervision and evaluation)

   चौकशी करा:तुमच्या कल्पना काय आहेत/धोरणे काय असू शकतात? या कल्पना/धोरणे कोण प्रत्यक्षात आणेल? कसे (विशेष उल्लेख केलेल्या धोरणाच्या अंमलबजावणीचे तपशील)?(प्रशिक्षणानंतरचे संनियंत्रण, पर्यवेक्षण आणि मूल्यमापन या विषयी त्यांची मते जाणून घेण्यासाठी)

- **Ask if they would like to add further comments**
- **Bring the meeting to a close by summarizing the main points**
- **Do not forget to say thank you to the participants for their time and active participation.**

FOCUS GROUP DISCUSSION GUIDE-COMMUNITY MEMBERS

I. CHARACTERISTICS OF THE PARTICIPANTS

Facilitators need to fill out the FGD log before the start of the FGD.

II. AVAILABILITY AND ACCEPTABILITY OF ANM:

1. We would really like to know about ANMs in your area – specifically we are interested in what works well with them and what works less well. To start with, can you describe any challenges or barriers that prevent people in your village from approaching an ANM?

   तुमच्या भागातील ANMs विषयी माहिती करून घ्यायला आम्हाला खरोखर आवडेल - विशेषतः, त्यांच्या बाबतीत काय फायद्याचे ठरते, आणि काय कमी फायद्याचे ठरते, हे तुमच्याकडून जाणून घ्यायला आम्हाला आवडेल. सुरुवातीला ANM कडे जाण्यापासून तुमच्या गावातल्या लोकांना कोणती आव्हाने किंवा अडथळे रोखतात, हे तुम्ही सांगू शकाल का?

   Prompt if not mentioned – availability of ANM, accessibility, availability of equipment and supplies, commodities, linkage with health centres, financial constraints

   जर त्यांनी उल्लेख केला नाही, तर तुम्ही **आधाराचे मुद्दे** द्या- ANMची उपलब्धता, तिच्या पर्यंत पोचण्याची मुभा, उपकरणे आणि वैद्यकीय साधनसामग्रीची उपलब्धता, आरोग्य केंद्रांशी दुवे, आर्थिक चणचण.

2. What are your recommendations for overcoming these barriers?

   या अडथळ्यांवर मात करण्यासाठी तुम्ही काय काय शिफारसी कराल?

3. Where or from whom do people in the community tend to seek information on health, nutrition, hygiene? (e.g. health centre, family, friends, ANM, radio, etc.)

   आरोग्य, पोषण, स्वच्छता या विषयीची माहिती तुमच्या समुदायातले लोक कुठून आणि कुणाकडून मिळवतात? (उदा. आरोग्य केंद्र, कुटुंब, मित्रमंडळी, ANM, रेडिओ इ.)

4. To what extent do people in the village seek advice or information from ANMs?

   ANMs कडून लोक किती प्रमाणात(कुठवर) सल्ला किंवा माहिती मिळवतात?

5. What sort of advice or information do people tend to ask from an ANM?

   ANM कडून लोक कशा प्रकारचा सल्ला किंवा माहिती मिळवतात?

6. We are interested in knowing what the community thinks about the ANMs – can you tell us your views about how acceptable the ANMs is locally? Are they useful to your community? In what aspect?

ANMs विषयी तुमचा समुदाय काय विचार करतो, हे जाणून घेण्यात आम्हाला रस आहे- स्थानिक पातळीवर ANMsना कितपत स्वीकारले जाते, हे तुम्ही सांगू शकाल का? तुमच्या समुदायासाठी त्या उपयुक्त आहेत का? कशा प्रकारे?

Prompt: Do you use ANM services for any of the following: ANC, child birth, PNC, family planning, child health, other primary health care?

आधाराचे मुद्दे:पुढील पैकी कोणत्याही बाबीसाठी तुम्ही ANMच्या सेवांचा उपयोग करता का?: ANC, प्रसूती, कुटुंब नियोजन, मुलांचे आरोग्य, अन्य प्राथमिक आरोग्य सेवा.

III. ANM'S KNOWLEDGE, ATTITUDE AND PRACTICES:

1. What do you think about the level of knowledge, and skillsof the ANMs providing services to your community? (For instance, ANC, delivery, PNC, family planning, child health, primary health care)

   तुमच्या समुदायाला सेवा पुरविणाऱ्या ANMsच्या ज्ञानाच्या आणि कौशल्यांच्या पातळीबद्दल तुम्हाला काय वाटते? (उदा. ANC, प्रसूती, PNC, कुटुंब नियोजन, मुलांचे आरोग्य, प्राथमिक आरोग्य सेवा)

2. In what areas do you think that they need to improve in terms of knowledge and skills to provide quality health services? Why?

   दर्जेदार आरोग्यसेवा पुरविण्यासाठी, ज्ञान आणि कौशल्यांचा विचार करता ANMsनी कोणत्या बाबतीत सुधारणा करणे आवश्यक आहे, असे तुम्हाला वाटते? का?

3. In which area of services would you like ANMs to improve? Why?

   आरोग्यसेवेच्या कोणत्या क्षेत्रात ANMs नी सुधारणा करावी, असे तुम्हाला वाटते? का?

4. How do you find the ANMs' attitude in service provision in your community?

   तुमच्या समुदायामध्ये सेवा पुरवीत असताना ANMsची वृत्ती (वागणे-बोलणे) तुम्हाला कसे वाटते?

5. In what areas do you think that ANMs should improve? Why?

   कोणत्या बाबतीत ANMsनी सुधारणा करावी, असे तुम्हाला वाटते? का?

- **Ask if they would like to add further comments**
- **Bring the meeting to a close by summarizing the main points**
- **Do not forget to say thank you to the participants for their time and active participation.**
