# Supplementary material for: Factors governing the performance of Auxiliary Nurse Midwives in India: A study in Pune district
Source: PLoS One. 2019 Dec 27;14(12):e0226831. doi: 10.1371/journal.pone.0226831 (PMC6934276; doi:10.1371/journal.pone.0226831)
Supplement: S3 Guide — (PDF) [file pone.0226831.s005.pdf]

OBSERVATION GUIDE

I. Background

- Date of observation:
- Time: Start (- -: - -), End (- -: - -)
- Location:
- The reference code for ANM being observed:
- Name of researcher completing the guide:
- Other researchers present:

II. Plans of activity to observe

- Type of ANM activity(ies): ANC, PNC, Family Planning counselling, Immunization etc.
- Context notes (Presence of support workers and other health workers such as ASHAs, weather, physical environment, etc.)

III. Activity[1]

- Essential clinical skills
- Communication and counselling during ANC, PNC, Family Planning, immunisation, etc.
- Managerial skills such record keeping, conducting meetings, organizing Village Health and Nutrition Day, etc.

IV. Post observation reflections

[1] ANM's job description as a standard
